# The Antistaphylococcal Activity of Citropin 1.1 and Temporin A against Planktonic Cells and Biofilms Formed by Isolates from Patients with Atopic Dermatitis: An Assessment of Their Potential to Induce Microbial Resistance Compared to Conventional Antimicrobials

**DOI:** 10.3390/ph9020030

**Published:** 2016-05-25

**Authors:** Malgorzata Dawgul, Wioletta Baranska-Rybak, Lidia Piechowicz, Marta Bauer, Damian Neubauer, Roman Nowicki, Wojciech Kamysz

**Affiliations:** 1 Faculty of Pharmacy, Medical University of Gdansk, Al. Gen. J. Hallera 107, 80-416 Gdansk, Poland; bauerm@gumed.edu.pl (M.B.); dneu@gumed.edu.pl (D.N.); kamysz@gumed.edu.pl (W.K.); 2 Faculty of Medicine, Medical University of Gdansk, Debinki 7, 80-211 Gdansk, Poland; wiolabar@gumed.edu.pl (W.B.-R.); lidiap@gumed.edu.pl (L.P.); rnowicki@gumed.edu.pl (R.N.)

**Keywords:** atopic dermatitis, antimicrobial peptides, biofilm, *Staphylococcus aureus*, superantigens, microbial resistance

## Abstract

*Staphylococcus aureus* (SA) colonizes the vast majority of patients with atopic dermatitis (AD). Its resistance to antibiotics and ability to form biofilms are the main origins of therapeutic complications. Endogenous antimicrobial peptides (AMPs) exhibit strong activity against SA, including antibiotic resistant strains as well as bacteria existing in biofilm form. The purpose of the present work was to determine the antistaphylococcal activity of two amphibian peptides against SA isolated from patients with AD. The AMPs demonstrated permanent activity towards strains exposed to sublethal concentrations of the compounds and significantly stronger antibiofilm activity in comparison to that of conventional antimicrobials. The results suggest the potential application of amphibian AMPs as promising antistaphylococcal agents for the management of skin infections.

## 1. Introduction

The skin serves as the first line of defense against infection by reducing microbial adherence and invasion [1]. In atopic dermatitis (AD), a chronic, relapsing inflammatory skin disease, the protective epidermal barrier is significantly compromised [2,3]. Both the etiology and pathogenesis of AD are very complex in nature. The disease is caused by the interplay of various factors, such as genetically related disturbances of both the structure and function of the epidermal barrier, allergic and immunologic abnormalities as well as the surrounding environment and infectious determinants such as *Staphylococcus aureus* (SA) [4].

SA is the major cause of skin and soft tissue infections which, despite the continuous development of medicines, are still difficult to treat [5,6]. Therapeutic problems are caused by the development of strains with reduced susceptibility to many antibiotics, such as methicillin-resistant SA (MRSA) [7]. SA is also a medically significant biofilm former. In terms of infections, these complex sessile microbial communities can grow on medical devices or host tissues. The process of biofilm formation is controlled by the accessory gene regulator (*agr*) quorum-sensing system which is one of SA’s virulence factors [8]. Due to their recalcitrance to host defense mechanisms and antimicrobial therapy, the biofilm-related infections move towards chronic disease status and reoccur after the withdrawal of antimicrobials [9,10].

Patients with AD have a predisposition towards frequent cutaneous infection with SA, *C. albicans* or the herpes simplex virus. Actually, over 90 percent of patients with AD have shown colonization with SA. More than half of the SA strains isolated from AD skin lesions have been shown to secrete superantigenic exotoxins, primarily SEA, SEB, and TSST-1, that stimulate marked activation of T cells and macrophages and presumably contribute to the exacerbation of AD [11,12].

Recently, the mechanisms of increased vulnerability to skin infections have been an area of intensive investigation. It has been postulated that the innate immune defense system of atopic skin based on naturally occurring antimicrobial peptides (AMPs) fails to restrict the growth of the organisms [3,13,14,15]. In view of the increasing microbial resistance to traditional antibiotics, AMPs are considered a source for the development of a novel antistaphylococcal therapy for patients with AD. Peptides secreted by the skin of amphibians constitute one of the best-studied groups of AMPs. Citropin 1.1 is a basic, highly hydrophobic, 16-amino acid peptide, produced by the dorsal and submental glands of the green tree frog *Litoria citropa*. It is one of the simplest broad-spectrum amphibian AMPs reported to date. Its antimicrobial potential towards a wide range of microorganisms has been established [16]. A broad spectrum of antimicrobial activity has also been displayed by temporin A, a basic, highly hydrophobic, 13-amino acid peptide derived from the frog *Rana temporaria* [17]. In the present study, we determined the activity of these two amphibian peptides and conventional antistaphylococcal antibiotics towards strains isolated from patients with AD. A characterization of SA isolates according to their variant of *agr*, their ability to produce superantigens, their development of resistance to antimicrobials and their biofilm formation has been performed.

## 2. Results and Discussion

### 2.1. Results

#### 2.1.1. SA Isolates

Among 15 examined patients with AD, 12 were colonized by SA. This corresponds to over 80 percent of the tested subjects. Nine strains showed the ability to produce superantigens (Table 1). Five SA isolates secreted staphylococcal enterotoxin A (SEA). C (SEC) and D (SED) were produced by two and three strains respectively, wherein one of these isolates secreted both toxins simultaneously.

None of the SA strains showed the ability to produce staphylococcal enterotoxin B or a toxin of staphylococcal shock syndrome TSST-1. The results obtained using toxin test kits were consistent with those obtained by PCR. All tested isolates carried the accessory regulator gene (*agr*). *Agr* types I and III were identified for six and five strains, respectively. Only one strain turned out to be *agr* II, while *agr* IV was missing.

#### 2.1.2. Activity against Planktonic Cells

The most potent antimicrobial agent was fusidic acid with MICs of 0.0625–0.125 mg/L against 11 tested strains. One isolate (SA 11) showed a reduced susceptibility to the compound (MIC = 4 mg/L). Erythromycin and mupirocin also inhibited staphylococcal growth in the majority of the strains at low concentrations (MICs below 1 mg/L). However, we have identified three and four isolates resistant to erythromycin (SA 2, 7, 8) and mupirocin (SA 4, 8, 9, 10), respectively. Linezolid was active at concentrations of 1–2 mg/L towards all SA isolates.

The amphibian peptides showed antimicrobial activity following a single application at higher concentrations. The MICs obtained for the majority of strains were 4 mg/L and 16 mg/L for citropin 1.1 and temporin A, respectively (Table 2).

**Figure 1:**
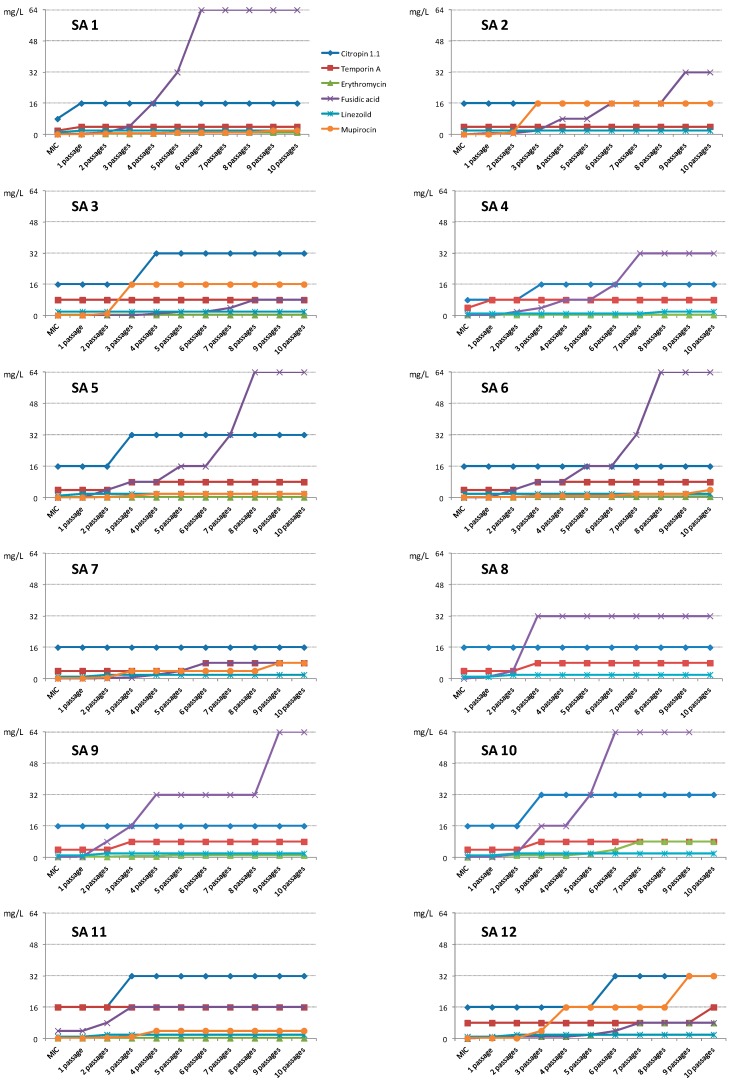
Changes in the initial activity of the compounds (MIC_0_) after 10 subsequent passages (MIC_1–10_) of exposure of clinical SA isolates to the compounds (0.5 × MIC). For fusidic acid and SA 10, MIC_10_ (128 mg/L) is not shown. Data are not shown for erythromycin (strains SA 2, 7 and 8) and mupirocin (strains SA 4, 8, 9 and 10)—the compounds were inactive towards the isolates at the tested concentrations (512 mg/L).

The minimum inhibitory concentration was determined for each strain after serial passages in a medium supplemented with the compounds below their active concentrations (0.5 × MIC). After 10 consecutive passages with the AMPs, the SA strains did not show any significant reduction in their susceptibility to AMPs. For these compounds, the active concentration after 10 passages either remained at the same level or was twice as high as the initial MIC (Figure 1, Table 2).

Linezolid also did not induce resistance in tested SA strains; the MIC_10_ were equal or twice as high as the initial MICs. No significant reduction in activity was observed also for erythromycin. For SA 3, 4, 5 and 11, the MIC did not change during the study, while for SA 1, 6 and 9 it increased 2–4 times. After 10 passages of isolates SA 10 and 12, their susceptibility was reduced 32 and 64 times in comparison to that of the initial MICs.

During this test with mupirocin, a significant decrease in antistaphylococcal activity was observed. For the strains SA 1, 5, 6, 7 and 11, which were very sensitive to the compound in the first MIC test, the active concentrations increased 16–32 times after the tenth passage. The strains SA 2, 3 and 12 reduced their susceptibility 128 times (Figure 1, Table 2).

An even more significant reduction in the susceptibility of the isolates was demonstrated by strains cultured in a medium supplemented with sublethal concentrations of fusidic acid. In this case, after the exposure of the strains to the compounds following the last passage, SA 11 and 7 were inhibited by the compound at concentrations 4 and 64 times higher than that of the initial MICs, respectively. SA 1, 2, 3, 4, 8 and 12 reduced their susceptibility 128–512 fold, while for the remaining strains MIC_10_ it was over 1000 times higher than that of MIC_0_ (Figure 1, Table 2).

#### 2.1.3. Activity against Staphylococcal Biofilm

Conventional antimicrobials demonstrated rather poor or no antibiofilm activity against the isolates. The least effective agent was erythromycin, which did not exhibit activity against biofilms formed by the majority of the SA isolates at the tested concentrations. Biofilm formed by the remaining strains (SA 3, 5, 10 and 11) was eliminated after exposure to erythromycin at a concentration over 2000 times higher than that of MICs.

Mupirocin was slightly more effective. For the most susceptible strains (SA 3 and 11), the elimination of the structures was noticed after using the antibiotic at a concentration of 128 mg/L, which was equal to (512–1024) times those of the MICs obtained for those strains. Biofilms formed by strains SA 3,6 and 12 were eradicated by mupirocin at 512 mg/L, while the remaining strains were not susceptible to the compound at the applied concentrations (over 2000 times that of the MICs).

Fusidic acid was inactive against the biofilm cultures of SA 1–4 and 7. SA 5, 6 and 8 were eliminated after application of the compound at concentrations of 128–512 mg/L (over (2–8) thousand × MIC). Biofilms formed by SA 9–12 were the most susceptible—the obtained MBEC ranged from 16 to 32 mg/L.

**Figure 2:**
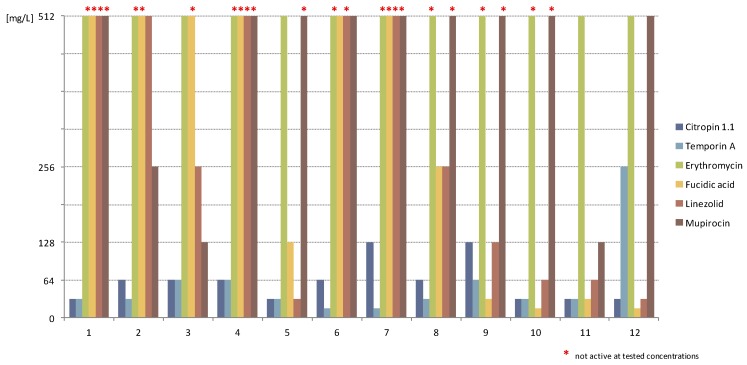
Antistaphylococcal activity of tested compounds towards biofilms formed by tested SA strains (mg/L); *: compound not active at the tested concentrations.

The same strains (SA 9–12) as well as SA 5 were the most susceptible to linezolid (MBEC 32–128 mg/L). SA 2 and 3 were eradicated at MBECs of 256–512 mg/L, whilst the remaining strains formed structures unsusceptible to the compound at the applied concentrations. The amphibian peptides showed markedly higher antibiofilm activity against all tested SA isolates. Temporin A eliminated the majority of the structures at concentrations (2–8) times those of the MICs. For strains SA 1, 4, 9 and 12, the effective concentrations towards biofilms (32–256 mg/L) were (16–32) times higher in comparison to the activity against planktonic cells. With citropin 1.1, the majority of the MBECs (16–32 mg/L) corresponded to (2–4) times those of the MICs, while biofilms formed by the strains SA 4, 7 and 9 were eradicated after application of citropin 1.1 at concentrations 8 times higher than those of the MICs (Table 2, Figure 2).

### 2.2. Discussion

According to previous studies, SA has been isolated from 75 to 90% of AD patients. This is compatible with our results. SA was found in the skin samples of 80% of the examined patients. Colonization with SA has been well documented as an exacerbating factor in AD [3,18,19,20]. According to the literature, more than half of the SA strains isolated from AD patients possess the ability to produce superantigens, and the prevalence rate is significantly higher than in the control groups (less than 5% of patients). In our study, 75% of the strains produced superantigens, which is consistent with the previous findings. Recent data suggest that SEA, SEB and TSST-1 are the most abundant toxins found in those strains. Four of the nine superantigen positive strains tested in the present work secreted SEA, whereas neither SEB nor TSST-1 were identified.

According to Lee *et al.*, SEA and SEB play a significant role in the initiation of atopic skin inflammation [2,12]. It has been proposed that toxins may stimulate TNF-α and IL-1α release from keratinocytes, macrophages and Langerhans cells. It has also been found that the majority of patients with AD possess circulating IgE specific to SEA, SEB and TSST-1, so the toxins are likely to function as allergens [21]. It has been established that the expression of SEA is not regulated by the accessory gene regulator *agr*, unlike SEB, SEC and SED [22]. In our study, the SA strains producing SEA belonged to *agr* group III. For isolates secreting SEC or SED, *agr* group I was identified. One strain belonged to *agr* group II and showed the ability to produce two toxins simultaneously (SEC and SED).

The *agr* locus of SA is described as a quorum-sensing gene cluster and may be a crucial regulator of bacterial cellular metabolism. Numerous studies demonstrate that the vast majority of clinical SA strains belong to *agr* group I. Among the MRSA isolated around the world, *agr* group III has often been identified. In our study, we found six, one and five strains belonging to *agr* groups I, II and III, respectively [23]. Strains representing *agr* type II showed a reduced susceptibility to β-lactams and glycopeptides [24].

The influence of the ability to produce staphylococcal enterotoxins on the activity of various antibiotics has previously been suggested. Superantigen-positive isolates have demonstrated a reduced susceptibility to chloramphenicol, ciprofloxacin, clindamycin and erythromycin [25]. Our study was conducted on a small population of SA strains and therefore, no statistical analysis in order to determine a relationship between the secretion of enterotoxins or the *agr* group and antibiotic effectiveness could have been performed. However, all the isolates demonstrating resistance to erythromycin belonged to *agr* group III and produced SEA. Also, two SA strains showing resistance to mupirocin were of *agr* type III and secreted SEA. Other mupirocin-resistant isolates were of *agr* type I; one produced SED, whilst the other did not secrete superantigens.

The type of *agr* has been reported to influence the ability of SA to form biofilms. Mirani *et al.* found that among biofilm positive methicillin sensitive SA (MSSA) strains (only 13.7% of all MSSA strains), *ca.* 50% carry *agr* type II, while 43.7% and 6.2% belong to group *agr* type I and 6.2% carry type III, respectively. The vast majority (81%) of biofilm negative MSSA isolates belong to group *agr* type I; 16% carry *agr* type III and the remaining 3% carry *agr* type II [23]. These data suggest that SA isolates from *agr* group I are not expected to be strong biofilm formers. In our study, SA 3, 5 and 9–12 indeed formed structures more susceptible to the majority of the antimicrobials in comparison to other strains.

The overuse of antibiotics is the main factor responsible for the high prevalence of bacterial strains resistant to antimicrobials. A significant decrease in susceptibility to erythromycin has often been reported over recent years [26]. In our study of three SA isolates, the lack of activity of erythromycin towards planktonic cells was noticed. However, the remaining strains showed a rather low rate of resistance development during the multiple passages of isolates in a medium supplemented with sublethal concentrations of those compounds.

Four of the 12 tested parental strains were not inhibited once exposed to mupirocin at the tested concentrations. After the tenth passage, the remaining isolates reduced their susceptibility significantly in comparison to that of the parental strains. Mupirocin is an approved antibiotic for topical use in the management of MRSA skin infections and is recommended for the eradication of SA carriage [27,28]. The high risk of reduction in its activity against SA has previously been debated. Long-term use of mupirocin induces the development of resistant strains. Some studies have revealed the colonization of patients with mupirocin-resistant strains without its previous widespread use, while others have demonstrated effective use of the antibiotic without a reduction in its activity [29,30,31].

A major decrease in activity was observed for fusidic acid, which proved to be the most active antibiotic in the initial MIC test. The compound has frequently been used in dermatology in the treatment of infected AD, and has a proven track record of efficacy [32]. Fusidic acid resistant SA has been reported all over the world, and its prevalence is higher in dermatology patients in comparison to other disciplines [33].

All the tested parental isolates were also susceptible to linezolid, and only an insignificant loss in its activity after consecutive passages was observed. According to the literature, the resistance rate of SA to this antimicrobial is low and estimated to be less than 1% [34].

Due to their mechanism based on interactions with the microbial cell plasma membrane [35], AMPs are believed not to induce bacterial resistance. This hypothesis was confirmed by several *in vitro* studies. Lipopeptide Laur-KK-NH_2_ proved to reduce the ability of *E. faecalis* strains to develop resistance to daptomycin [36]. The resistance of *P. aeruginosa* to a panel of eight peptides was increased by no more than fourfold after 30 passages in media supplemented with peptides below their MIC [37]. Our study provided corresponding results—the susceptibility of clinical isolates to amphibian peptides was reduced twofold as compared to that of the initial strains, or remained unchanged.

The unique mechanism of action also determines the activity of AMPs towards microbial biofilms. The compounds have proved to be active *in vitro* and *in vivo* against multidrug resistant bacteria in planktonic forms as well as towards biofilms at various stages of their development [38,39,40,41,42,43,44,45]. In the present study, the conventional antimicrobials demonstrated rather low antibiofilm activity, whilst AMPs were effective towards all SA isolates. Bacteria in biofilm form are frequently reported to exhibit tolerance to antimicrobials, while the same cells are sensitive in the planktonic state. This resistance/persistence of biofilm is directly related to low metabolic activity and the slow growth rate of bacteria in such a community. According to the mechanism of action, based on interactions with the microbial cell membrane, AMPs can act on both slow-growing and non-growing bacteria [46].

Human β-defensins HB43, HB55 and HBPM4 have demonstrated activity towards MRSA [38]. Human β-defensin 3 (HBD-3) was also an effective agent against *S. aureus* in biofilm form [39]. The antistaphylococcal activity of human cathelicidin LL-37 and its analogues has also been extensively investigated. LL-37 disrupted the formation of biofilm and eradicated the pre-grown biofilms of *P. aeruginosa*. LL-37 also inhibited the attachment and development of the biofilms of *S. epidermidis* [40,41].

In previous studies, amphibian peptides, aurein 1.2 and uperin 3.6 demonstrated strong antibacterial activity towards Gram-positive nosocomial cocci [42,43]. Citropin 1.1 demonstrated synergic action with rifampin and minocycline against an *S. aureus* biofilm [44]. Temporin A also proved its effectiveness against biofilms formed by gram-positive strains [45]. The results of the present study confirm the strong antibiofilm potential towards SA in comparison to that of conventional antimicrobials, which exhibited very poor antibiofilm activity. This is compatible with our previous study, where those antimicrobials were ineffective against structures formed by SA isolated from patients suffering from staphylococcal skin infections. The results obtained for conventional antistaphylococcal agents are also in accordance with data in the literature reporting that biofilm-associated bacteria exhibit inherent tolerance to antimicrobial compounds and that the MBECs are over 1000 fold higher in comparison to those of the MICs [47].

Both citropin 1.1 and temporin A have proved not only to be very potent antimicrobials, but also to be non-toxic towards human keratinocytes. In our previous study, the compounds did not exert any toxic effect on HaCaT cells at their microbiologically active concentrations and presented the most promising activity *vs.* toxicity ratio [48]. Therefore, the AMPs studied could be considered for treatment of skin infections, both on intact and abraded skin.

## 3. Experimental Section

### 3.1. Peptides

The compounds included in this study, citropin 1.1 and temporin A (Table 3), were synthesized manually using Fmoc chemistry on polystyrene resin that had been modified by the Rink amide linker [49]. Deprotection of the Fmoc group was carried out in a 20% solution of piperidine (Merck, Darmstadt, Germany) in dimethylformamide (DMF, Honeywell, Seelze, Germany). After washing the resin with DMF and dichloromethane (DCM, Honeywell), the amino acids were coupled using the DMF/DCM (1:1, *v*/*v*) mixture in the presence of coupling agents such as 1-hydroxybenzotriazole (HOBt; Orpegen, Heidelberg, Germany) and diisopropylcarbodiimide (DIC; Peptideweb, Zblewo, Poland). The extent of both the deprotection and acylation was monitored by the chloranil test. The peptides were cleaved from the resin with trifluoroacetic acid (Apollo Scientific, Denton, UK), water, triisopropylsilane and phenol (Sigma Aldrich, St. Louise, MO, USA) in the ratio 92.5:2.5:2.5:2.5 *v*/*v*, precipitated in cold diethyl ether (Chempur, Piekary Slaskie, Poland) and lyophilized. All crude products were purified by reversed-phase high performance liquid chromatography (RP-HPLC) in a gradient of acetonitrile (Sigma Aldrich)—water. The confirmed purity of both compounds was over 97 percent. The identity of the peptides was confirmed by matrix-assisted laser desorption/ionization time-of-flight-mass spectrometry (MALDI-TOF-MS). 

### 3.2. Bacterial Isolates

Fifteen patients diagnosed with staphylococcal skin infections and hospitalized at the Dermatological Clinic of the Medical University of Gdansk, were enrolled in the study. Skin swabs were taken from each patient. SA was identified using standard previously described microbiological procedures [47].

#### Superantigen Detection

A Staphylococcal Enterotoxin Test Kit (SET-RPLA KIT TOXIN DETECTION KIT, Oxoid, Basingstoke, UK) was used for the detection of staphylococcal enterotoxins A, B, C and D, and a staphylococcal toxic shock syndrome toxin kit (TST-RPLA TOXIN DETECTION KIT, Oxoid) was employed for the detection of STSS in a culture, using reversed passive latex agglutination.

### 3.3. Toxin Gene Detection Using the PCR Technique

DNA was isolated in accordance with Barski *et al.* [50]. DNA amplification was carried out in a Perkin Elmer 2400 Thermocycler (Perkin Elmer Corporation, Norwalk, CT, USA). The primers for enterotoxins (SEA, SEB, SEC and SED) and the TSST-1 toxin were used as described previously [51]. DNA amplification was carried out with the following thermal cycling profile: an initial denaturation at 94 °C for 5 min was followed by 35 cycles of amplification (denaturation at 94 °C for 2 min, annealing at 57 °C for 2 min, and an extension at 72 °C for 1 min) and a final extension at 72 °C for 7 min. The PCR products were analyzed on a 2% agarose gel (Sigma Aldrich, St. Louise, MO, USA) in the presence of ethidium bromide under UV illumination. Control strains for the PCR detection of virulence factor genes were obtained from the National Institute of Public Health (NIPH; Warsaw, Poland).

### 3.4. Identification of S. Aureus agr Groups I, II, III, IV

DNA was isolated in accordance with Barski *et al.* [50]. Clinical *S. aureus* isolates were studied with the use of the PCR, using *agr* groups I, II, III, and IV-specific primers reported elsewhere [51]. The PCR program consisted of an initial denaturation step at 94 °C for 5 min, 30 cycles of denaturation at 94 °C for 1 min, annealing at 55 °C for 1 min, an extension at 72 °C for 1 min and a final extension step at 72 °C for 0.5 min. The products were analyzed using the standard methods of agarose gel electrophoresis and UV detection.

### 3.5. Antimicrobial Assay

The minimum inhibitory concentration (MIC) and minimum biofilm elimination concentration (MBEC) of the peptides were determined using a microbroth dilution method with the Mueller Hinton Broth II (MHB II; Becton Dickinson, Heidelberg, Germany). Bacteria at initial inoculums of 5 × 10^5^ CFU/mL were placed on polypropylene 96-well plates and exposed to tested compounds at graded concentrations for 18 h at 37 °C. The MIC was taken as the lowest drug concentration at which a noticeable growth was inhibited. Biofilms formed by clinical SA strains were cultured on polystyrene 96-well plates (Kartell, Noviglio, Italy). Bacteria at initial inoculums of 5 × 10^8^ CFU/mL were added to the plates and incubated at 37 °C. After 72 h, the biofilms were exposed to tested compounds for 24 h. Resazurin (Sigma Aldrich, St. Louise, MO, USA) was added as a cell-viability reagent. MBEC was taken as the lowest drug concentration at which the reading was compatible with the negative control (sterile MHB II with resazurin).

Ten consecutive passages of SA strains in a medium supplemented with tested compounds were performed in order to evaluate the progressive reduction in susceptibility to the tested compounds. After each passage, an MIC determination with the above described microbroth dilution method with MHB II was performed.

Identical tests were performed for conventional antimicrobials: erythromycin, fusidic acid, mupirocin (Sigma Aldrich, St. Louise, MO, USA) and linezolid (kindly provided by Pfizer). The experiments were performed in triplicate.

## 4. Conclusions

The AMPs exhibited activity against all SA strains isolated from patients with AD, while several strains were resistant to conventional antistaphylococcal agents. In general, the activity of antibiotics against the majority of the isolates in their planktonic form was significantly higher in comparison to that of the AMPs. However, the effectiveness of the most potent conventional antimicrobials, such as mupirocin or fusidic acid, was decreasing gradually during the multiple passages in the medium supplemented with the subinhibitory concentrations of the compounds. A significantly lower ability to induce bacterial resistance was observed for linezolid and the AMPs. Citropin 1.1 and temporin A also proved to be potent antibiofilm agents, unlike conventional antibiotics. The antimicrobials used in the management of staphylococcal infections either exhibited poor activity against structures grown on polystyrene surfaces or turned out to be inactive at the tested concentrations.

Taking under consideration the role played by *S. aureus* in etiopathogenesis in AD and its growing resistance to conventional antibiotics, the development of new therapeutic agents is crucial for modern medicine. Based on the literature data and the obtained results, AMPs seem to be highly promising candidates for the management of staphylococcal skin infections.

## Figures and Tables

**Table 1:** Characterization of SA strains acquired from patients with AD, with regard to their ability to produce staphylococcal toxins and *agr* type.

Strain No.	Toxins					*Agr*
	SEA	SEB	SEC	SED	TSS	
1	SEA	-	-	-	-	III
2	SEA	-	-	-	-	III
3	-	-	-	-	-	I
4	SEA	-	-	-	-	III
5	-	-	-	-	-	I
6	-	-	SEC	SED	-	II
7	SEA	-	-	-	-	III
8	SEA	-	-	-	-	III
9	-	-	-	SED	-	I
10	-	-	-	-	-	I
11	-	-	SEC	-	-	I
12	-	-	-	SED	-	I

**Table 2:** Antistaphylococcal activity against planktonic cells of parental SA clinical isolates (MIC), strains subjected to 10 passages in a medium supplemented with antimicrobials (MIC_10_) and bacteria growing in a biofilm form (MBEC) (mg/L).

Compound/SA strain	SA	1	2	3	4	5	6	7	8	9	10	11	12
Citropin 1.1	MIC	8	16	16	8	16	16	16	16	16	16	16	16
	MIC_10_	16	16	32	16	32	16	16	16	16	32	32	32
	MBEC	32	64	64	64	32	64	128	64	128	32	32	32
Temporin A	MIC	2	4	8	4	4	4	4	4	4	4	16	8
	MIC_10_	4	4	8	8	8	8	8	8	8	8	16	16
	MBEC	32	32	64	64	32	16	16	32	64	32	32	256
Erythromycin	MIC	0.25	>512	0.25	0.25	0.25	0.25	>512	>512	0.25	0.125	0.25	0.25
	MIC_10_	1	>512	0.25	0.25	0.25	0.5	>512	>512	1	8	0.25	8
	MBEC	>512	>512	512	>512	512	>512	>512	>512	>512	>512	512	512
Fusidic acid	MIC	0.125	0.125	0.0625	0.125	0.0625	0.0625	0.125	0.0625	0.0625	0.125	4	0.0625
	MIC_10_	64	32	8	32	64	64	8	32	64	128	16	8
	MBEC	>512	>512	>512	>512	128	512	>512	256	32	16	32	16
Linezolid	MIC	1	2	2	1	1	2	1	1	1	1	1	1
	MIC_10_	2	2	2	2	2	2	2	2	2	2	2	2
	MBEC	>512	512	256	>512	32	>512	>512	256	128	64	64	32
Mupirocin	MIC	0.125	0.125	0.125	>512	0.125	0.25	0.25	>512	>512	>512	0.25	0.25
	MIC_10_	2	16	16	>512	2	4	8	>512	>512	>512	4	32
	MBEC	>512	256	128	>512	>512	512	>512	>512	>512	>512	128	512

**Table 3:** Amino acid sequences of the synthesized AMPs.

Compound	Amino Acid Sequence
Citropin 1.1	GLFDVIKKVASVIGGL-NH_2_
Temporin A	FLPLIGRVLSGIL-NH_2_
